# Automatic longitudinal assessment of brain metastases improves detection of disease progression

**DOI:** 10.1093/noajnl/vdag036

**Published:** 2026-02-11

**Authors:** David Weiss, Khaled Bousabarah, Nazanin Maleki, Saahil Chadha, Divya Ramakrishnan, Ajay Malhotra, Ahmed W Moawad, Klara Osenberg, Spyridon Bakas, Veronica Chiang, Wondwossen Lerebo, Dirk Smeets, Elizabeth Schrickel, Fatima Memon, Sanjay Aneja, Cornelius Deuschl, Mingde Lin, Mariam Aboian

**Affiliations:** Department of Radiology and Biomedical Imaging, Yale School of Medicine, New Haven, CT, USA; Department of Diagnostic and Interventional Radiology and Neuroradiology, Essen University Hospital, Essen, Germany; Visage Imaging, GmbH, Berlin, Germany; Department of Radiology, Children’s Hospital of Philadelphia, Philadelphia, PA, USA; Department of Radiology, University of Pennsylvania, Philadelphia, PA, USA; Department of Radiology and Biomedical Imaging, Yale School of Medicine, New Haven, CT, USA; Department of Radiology and Biomedical Imaging, Yale School of Medicine, New Haven, CT, USA; Department of Radiology and Biomedical Imaging, Yale School of Medicine, New Haven, CT, USA; Division of Neuroradiology, Department of Radiology, Hospital of University of Pennsylvania, Philadelphia, PA, USA; Department of Radiology and Biomedical Imaging, Yale School of Medicine, New Haven, CT, USA; Department of Pathology and Laboratory Medicine, Indiana University School of Medicine, Indianapolis, IN, USA; Departments of Radiology and Imaging Sciences, Neurological Surgery, Biostatistics and Health Data Science, Indiana University School of Medicine, Indianapolis, IN, USA; Department of Computer Science, Luddy School of Informatics Computing and Engineering, Indiana University, Indianapolis, IN, USA; Department of Neurosurgery, Yale School of Medicine, New Haven, CT, USA; Department of Radiology, Children’s Hospital of Philadelphia, Philadelphia, PA, USA; icometrix, Leuven, Belgium; Department of Neuroradiology, Wexner Medical Center, Ohio State University, Columbus, OH, USA; Carolina Radiology, Myrtle Beach, SC, USA; Department of Therapeutic Radiology, Yale School of Medicine, New Haven, CT, USA; Department of Diagnostic and Interventional Radiology and Neuroradiology, Essen University Hospital, Essen, Germany; Department of Radiology and Biomedical Imaging, Yale School of Medicine, New Haven, CT, USA; Visage Imaging, Inc., San Diego, CA, USA; Department of Radiology, Children’s Hospital of Philadelphia, Philadelphia, PA, USA; Department of Radiology, University of Pennsylvania, Philadelphia, PA, USA

**Keywords:** artificial intelligence, brain metastasis, inter-rater variability, lesion tracking, treatment response assessment

## Abstract

**Background:**

Characterization of multiple brain metastases (BMs) during treatment is limited by lack of available tools for individual volumetric lesion tracking. This study investigated role of a Picture Archiving and Communication System (PACS)-integrated artificial intelligence (AI) prototype lesion tracking tool (AI-LTT) for segmentation, enabling automatic assessment of longitudinal lesion changes compared to manual analysis by 2 neuroradiologists, focusing on inter-observer variability.

**Methods:**

Longitudinal studies of patients with BMs who underwent stereotactic radiotherapy were assessed. In manual workflow, 2 board-certified neuroradiologists displayed current and up to 7 prior studies of each patient and measured orthogonal lesion diameters (DMAX) manually. In AI-LTT-assisted workflow, a custom hanging protocol automatically selected, displayed, and 3D registered T1 gadolinium-enhanced MR sequences in up to 8 studies. A Scalable and Transferable U-Net (STU-Net) trained on BraTS METS 2023 BM dataset was used for segmentations from which DMAX were extrapolated. Neuroradiologists revised AI measurements as needed. Time and number of mouse clicks for both workflows were recorded.

**Results:**

A total of 40 patients with 158 MRIs were investigated. Median DSC of STU-Net segmentations was 0.771. Intra-class and Spearman correlation coefficients of manual diameter measurements were 0.959 and 0.961, respectively. Out of 353 true lesions, 59 were detected by only one (R1) and 15 by only the second neuroradiologist (R2). Among 74 lesions discrepant between readers, 14 proceeded to complete response, 4 partial response, 39 stable disease, but 12 developed progressive disease.

**Conclusions:**

AI-assisted workflow for BM analysis improves follow up of lesions and has potential to improve detection of lesions that progress over time of treatment.

Key Points

- Real-time reads of small brain metastases (BM) by experienced neuroradiologists exhibit significant variability.
- Reporting variability of progressing BM can impact patient treatment.
- Prototype AI-LTT enhances neuroradiology workflow and improves consistency across timepoints.

Importance of the Study

Accurate assessment and reporting of brain metastases (BMs) over time are prerequisites for a successful treatment regimen but are laborious and exhibit inter-observer variability, which affects patient treatment. Characterizing BMs during neuro-oncological treatment, particularly smaller lesions, remains challenging due to the lack of dedicated tools. This study presents a Picture Archiving and Communication System (PACS)-integrated AI prototype lesion tracking tool (AI-LTT) for automated longitudinal BM segmentation and treatment response assessment. Substantial AI-LTT-facilitated improvements in workflow efficiency for lesion-based evaluation of BM magnetic resonance imaging workup with multiple prior comparison images were demonstrated, compared with the standard manual measurement workflow involving 2 board-certified neuroradiologists. Importantly, significant variability in real-time reads of small metastases by neuroradiologists was revealed, underscoring current challenges faced in clinical practice. Imaging-based assessment of individual metastatic lesion response determines patient’s treatment courses and is particularly important in progressive disease. AI-driven tools may reduce this discrepancy and enable standardized BM assessment and treatment on a lesion-level.

In adult patients, brain metastases (BMs) are the most common cerebral neoplasm as the annual incidence of more than 100,000 in the United States continues to rise.^1–3^ Magnetic resonance imaging (MRI) is the current standard of care for diagnosis and posttreatment follow-up of BM in clinical practice.^4^ Among several available therapeutic modalities for BM, stereotactic radiosurgery (SRS) has achieved widespread acceptance, presenting a targeted treatment approach, even in patients with multiple (>4) metastatic lesions.^2^^,^^5^^,^^6^ Due to the widespread acceptance of these personalized medicine techniques in the treatment of BM, treatment response assessment requires longitudinal lesion-based assessment, exact measurement techniques, and structured reporting on a lesion level for successful neuro-oncological treatment.^4^^,^^7^ Comprehensive lesion-based follow-up using multiple prior imaging studies has not been instituted in most clinical practices.

At present, the standard of care imaging includes 2-dimensional measurements in neuroradiological reports and clinical trials. Treatment response evaluation is based on response assessment in neuro-oncology brain metastases (RANO-BM) criteria.^8^ One of the major limitations of current reporting is the variability between the measurements of different readers and their experiences, which can raise confusion in oncologist interpretations. In clinical trials, specialized software is commonly used to perform these additional measurements that are usually not reported in clinical radiology reports. These additional measurements are usually funded through research funds and require data management infrastructure for the transfer of studies from the clinical interface into the research software. In addition to logistical limitations to performing detailed measurements, significant inter- and intra-rater variability limits the measurement of lesions smaller than 10 mm, which is why RANO-BM does not recommend measuring BM below this threshold. With a large proportion of BM being below this size, these criteria cannot be used for BM less than 10 mm, and thus, treatment response assessment cannot be quantitatively evaluated for these lesions. The current variability in BM measurement and lack of lesion-level tracking in routine practice can significantly impact treatment response assessment and affect treatment decisions, particularly for smaller lesions. Therefore, reproducible measurement techniques and reports are pivotal for accurate and consistent treatment.^7^^,^^9–12^

Manual intracranial lesion measurement without accessible measurement and organization tools is time-consuming, and in patients with multiple BMs, prolonged reading times due to measurement time requirements are common.^3^ Recent advances have resulted in improved survival and longer follow-up times, which increases the number of scans that would have to be compared for lesion-based treatment response assessment.^1^^,^^2^ This increasing workload may contribute to the rising burn-out rate among neuroradiologists and the emergence of generalized reports that do not provide lesion-based metrics.^13^ This presents an opportunity for automation and standardization of accurate measurement and longitudinal assessment of BM that can assist neuroradiologists but also provide contour outlines for radiation therapy planning, clinical trial response assessment, and improved communication with patients. Automated treatment response assessment could bypass laborious and repetitive measurements, allowing a focus on the actual core value of neuroradiologists—interpreting and communicating medical images.

Artificial intelligence (AI)-based models have shown effectiveness in high-precision automated BM segmentation and workflow acceleration in neuroimaging. However, few are available for diagnosing, monitoring, or planning therapies for patients with BM in clinical practice.^14–18^ Thus, the translation of published algorithms from the literature into a clinically usable application within the clinical environment still needs to be addressed.

In this study, we aimed to evaluate the role of an AI-based automated measurement prototype incorporated into the Picture Archiving and Communication System (PACS) in assessment of brain metastases over the course of treatment after stereotactic radiosurgery. We focused on evaluating inter-observer variability in board certified neuroradiologists and how it affects the final disease assessment in patients.

## Methods

### Dataset and Study Design

This retrospective single-center study was compliant with the Health Insurance Portability and Accountability Act and approved by the Institutional Review Board. Patients were randomly selected from a preexisting local database containing more than 7,000 patients with a confirmed clinical or pathological BM diagnosis. The inclusion criteria comprised patients with at least 3 available longitudinal MRI studies, known primary malignancy, and past SRS treatment at Yale New Haven Hospital. The exclusion criteria comprised studies with a slice thickness ≥2.5 mm to account for sub-centimeter lesions and studies with substantial movement artifacts. From 65 initially randomly selected patients, we identified 40 patients with 158 longitudinal studies who met our inclusion criteria. Per patient, we included 3 to 8 MRIs comprising either an SRS planning study with follow-up studies or follow-up studies only. The images were transferred from the clinical PACS to a research instance of the institution’s PACS (AI-Accelerator, Visage Imaging, Inc., San Diego, CA) and deidentified whereby images of the same patient were matched using the same de-identified patient identification number. Forty selected patients were randomly divided into 2 groups: Cohort A including 30 patients with 108 studies was used to validate the segmentation model; 10 patients with 50 studies (cohort B) were selected for model validation and workflow experiments. No image preprocessing or data manipulation of the raw clinical images was conducted; only T1 post-gadolinium sequences were investigated within our study. Baseline patient demographics and imaging characteristics were recorded.

### AI-Enabled Lesion Tracking Tool

**Figure 1:**
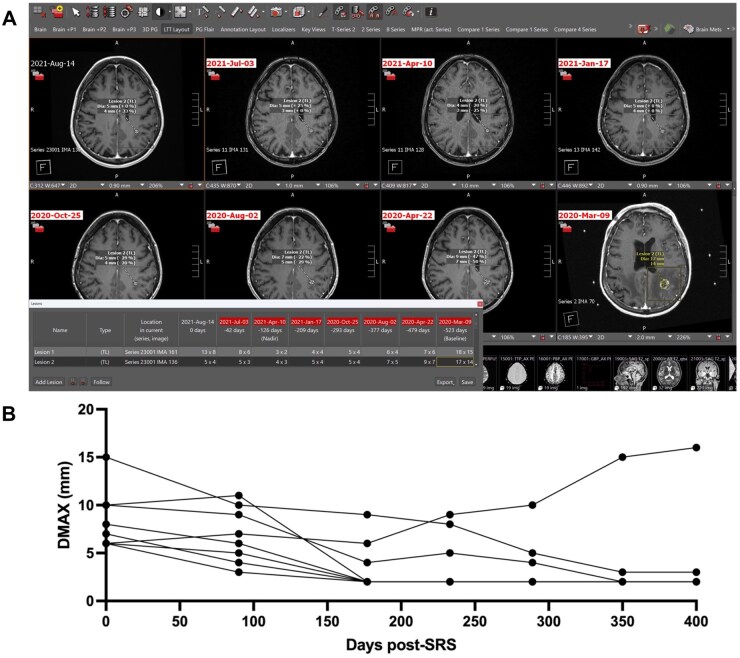
Layout of PACS-integrated AI-enabled lesion tracking tool and treatment-response curve. (A) Eight co-registered series of T1 gadolinium-enhanced sequences are depicted while the implemented hanging protocol and LTT layout feature are activated. Within the shown lesion tool card, 2 identified metastases and their AI-generated maximum orthogonal diameters, highlighted on each series, are matched, and longitudinally aligned. The AI-LTT provides automated critical information regarding the individual behavior in size and treatment response of each lesion over up to 8 study dates. (B) The treatment response curve of another patient, including 8 longitudinally tracked lesions, is presented.

The AI prototype lesion tracking tool (AI-LTT) is embedded within the AI-Accelerator and can operate on up to 8 studies. The previous version of this application, described by Cassinelli Petersen et al., was augmented with AI and validated on pre- and post-SRS MRI studies.^19^ Before deploying the tool, the user loads and opens the MRI studies of interest. An integrated custom hanging protocol automatically selects, displays, and 3D registers the T1 post-gadolinium sequences with the thinnest available slice thickness for each study chronologically on the screen, requiring one click on the “LTT-layout” button. Applying the “Segmentation to lesion” button, the integrated STU-Net algorithm is deployed. This model trained on the publicly available BraTS METS 2023 dataset automatically generates 3D segmentations of the enhancing metastatic tumors on all selected studies from which visible orthogonal maximum diameters (DMAX) are extrapolated in the axial plane. The segmented anatomically distinct lesions of each involved study are automatically matched and aligned over time; these metastatic tumors and their related orthogonal diameters are presented within an organizational lesion tool card. Selecting an identified lesion in this tool card, the interactive layout displays the orthogonal diameters on the related image slice and co-registers all other longitudinal studies showing the same slices of the anatomically distinct lesion. The DMAX of the selected lesion and the percent change of each diameter compared to the prior MRI are highlighted in yellow. The interactive layout allows manual refinements of the determined lesion diameters as well as the deletion and addition of lesions within the viewports, which are assigned to the tool card in real-time. This lesion tool card can be exported to the free-text MRI report as a CSV file containing the longitudinally aligned lesions, the related image series, orthogonal diameters, and the percent change compared to the prior study (Figure 1).

### Dataset Annotation and Definition of Ground Truth

Reference standard 3D segmentations of the enhancing tumors were performed on all included MRIs by a board-certified neuroradiologist. To curate the reference standard DMAX measurements for the inter-rater variability assessment, the results of the below-described workflow experiment were used: 2 additional independent board-certified neuroradiologists (Reader 1 [R1] and Reader 2 [R2]) measured DMAX of the lesions on the studies of cohort B separately. The DMAX measurements from R1 and R2 were averaged for each BM and represented the preliminary reference standard, whereby the third neuroradiologist verified the lesions on which the 2 readers disagreed and determined DMAX to define the final reference standard annotations.

### Validation of the Integrated Segmentation Network

To account for the accuracy of the AI-predicted measurements, we validated the performance of the implemented segmentation model in 2 distinct steps using all studies of cohorts A and B: Spatial segmentation and lesion detection. The overlap of the AI-generated and reference standard 3D segmentation masks was quantified for each study. To quantify the algorithm’s performance in detecting BM, the agreement of the reference standard and AI-generated annotations was evaluated. The AI-predicted false positive segmentation masks were visually analyzed to separate definite false positive lesions from predicted lesions that warrant a second review from a neuroradiologist for verification.

### Assessment of AI-LTT-Facilitated Efficiency Gains

We analyzed the efficiency gains provided by the tool for R1 and R2 separately, measuring the time and number of mouse clicks needed for each patient of cohort B. After training both readers in the measurement tools and the AI-LTT interface available on the AI-Accelerator, each reader measured the metastatic tumors of the longitudinal MRIs of each patient in the fully manual workflow. Here, the recording started as the related studies were selected and loaded; the reader manually displayed each study chronologically within an 8-viewer interface. Subsequently, the neuroradiologist read the MRIs concerning metastatic brain tumors using all available MRI sequences and measured the DMAX of each identified lesion manually with a caliper on T1 post-gadolinium sequences, which does not provide any measurement organization. After 10 days, these tasks were repeated by R1 and R2 using the same dataset and the AI-LTT assistance. Similarly, the recording started as the studies were selected and loaded. Applying the “LTT layout” button, the tool displayed T1 post-gadolinium sequences of each study chronologically in an 8-viewer interface, whereas the reader corrected the sequences as needed. Next, orthogonal BM diameters were generated by pushing the “Segmentation to lesion” button and the neuroradiologist read the images concerning metastatic tumors, verifying the displayed measurement predictions. Based on clinical judgment, the reader manually corrected, added, or deleted the AI-generated orthogonal diameters within the viewports as needed. In both experiments, the time recording stopped as all measurements were completed for each patient. In the AI-assisted workflow, R1 and R2 were blinded to their own prior manual measurements, that is, their measurements from the fully manual workflow were not visible. R1 and R2 were not blinded to the study purpose. For each reader, the manual and AI-assisted workflows were compared regarding the seconds and number of mouse clicks needed for each patient. In addition, the seconds and number of mouse clicks were correlated with the maximum number of lesions displayed on the images of each patient.

### Inter-Rater Variability of Manual BM Measurements

The measurements of R1 and R2 recorded in the manual workflow without AI assistance were investigated. Based on the final reference standard annotations, the performance in lesion detection was evaluated for both readers. The inter-rater variability of the readers’ DMAX measurements was assessed; here, lesions, that were detected by either R1 or R2 but not by both, were excluded. Moreover, clinically relevant characteristics of discrepant brain lesions were recorded, whereas modified RANO-BM criteria were applied to each individual lesion to assess the treatment response of the discrepant tumors. The observation endpoint was defined as 12 months after the first study, or the last available study if follow-up was shorter.

### Statistics

In this study, Shapiro-Wilk tests were used to investigate the normality of data. Normally distributed continuous data were reported using mean and standard deviation, otherwise median and inter-quartile range were documented; count and percentage were reported for categorical variables. Patient demographics and imaging characteristics were analyzed concerning differences between cohorts A and B by applying Mann-Whitney and Fischer’s exact tests. To validate the model’s performance in BM detection, precision, sensitivity, and F1-score were used as metrics. For the validation of the AI-predicted 3D segmentation masks, the patient-wise Sorensen-Dice Similarity Coefficient was calculated. Wilcoxon Signed-Rank tests were applied to compare the seconds and number of mouse clicks recorded in the manual and AI-assisted workflow. To account for the inter-rater variability captured in the manual workflow, the precision, sensitivity, and F1-score in lesion identification were calculated for R1 and R2. For manual DMAX measurements, Bland-Altman plots, the intra-class correlation coefficient, and the Spearman correlation coefficient were utilized. A *P* value <0.05 was considered statistically significant.

## Results

### Patient Demographics and Imaging Characteristics

Baseline patient demographics and imaging parameters were recorded to provide a comprehensive impression of the investigated patients and the underlying experimental setting. Patients involved in the workflow experiments (cohort B) had significantly more follow-up studies (*P* = 0.002) and lesions at the time of the initial study (*P* = 0.039) than patients of cohort A; significantly shorter echo times were observed in studies of cohort B (*P *= 0.001). Regarding all other recorded variables, neither group differed substantially from the other (Table 1). The images investigated in this study were acquired on several different MRI scanners (see Supplementary Material).

**Table 1:** Patient demographics and imaging characteristics

Characteristic	Cohort A (30 patients, 108 studies)		Cohort B (10 Patients, 50 Studies)		*P* value
	Median	IQR	Median	IQR	
Age (in years)	61	55-69	67	62-71	0.125
Survival after initial study (in days)	625	347-1239	1059	585-1703	0.414
Number of follow-up studies per patient	3	3-4	4	4-6	0.002
Number of lesions per patient at initial study	3	2-4	7	2-11	0.039
Average lesion size at initial study (in mm)	8.7	5.4-14.2	8.6	8.0-9.8	0.661
	Count	Percentage	Count	Percentage	
Sex					0.246
Male	8	26.7	5	50	
Female	22	73.3	5	50	
Race					0.442
White	29	96.7	9	90	
Black	0	0	1	10	
Indian or native American	1	3.3	0	0	
Survival status					0.999
Alive	14	46.7	5	50	
Deceased	16	53.3	5	50	
Primary cancer					0.835
NSCLC	9	30	5	50	
SCLC	2	6.8	1	10	
Breast-CA	9	30	1	10	
Melanoma	4	13.4	3	30	
Colorectal-CA	1	3.3	0	0	
Renal-CA	1	3.3	0	0	
Sarcoma	1	3.3	0	0	
Vulva-CA	1	3.3	0	0	
Endometrium-CA	1	3.3	0	0	
Oropharyngeal-CA	1	3.3	0	0	
Field strength					0.999
Proportion in cohort A					
1.5 T (in %)				72 (66.7)	
3 T (in %)				36 (33.3)	
Proportion in cohort B					
1.5 T (in %)				33 (66)	
3 T (in %)				17 (34)	
Parameter	Median		IQR		
Slice thickness (in mm)					0.956
Cohort A	1.0		1.0-2.0		
Cohort B	1.0		1.0-1.2		
Repetition time (in ms)					0.407
Cohort A	1900		13.0-1900		
Cohort B	1900		13.0-1900		
Echo time (in ms)					0.001
Cohort A	3.08		2.96-4.90		
Cohort B	2.96		2.46-3.08		

Clinical variables of all included 40 patients are presented. Relevant imaging parameters of the investigated MRI studies are displayed for cohorts A and B separately. Cohorts A and B were compared.

### Performance of STU-Net BM Segmentation Algorithm (AI-LTT) within PACS

A total of 607 cerebral metastases were investigated. In lesion detection, the precision and sensitivity were 0.924 and 0.827 for all lesions and 0.968 and 0.913 for lesions ≥5 mm; the precision was 0.824 for lesions <5 mm. A total of 82 false positive lesions were initially recorded. Here, we identified 41 AI-predicted BMs that warrant a second review from a neuroradiologist and 41 definite false positive predicted lesions, none of which were located outside the brain parenchyma. The segmentation network showed a median patient-wise Dice Similarity Coefficient of 0.771 [0.567-0.837] (Table 2).

**Table 2:** STU-Net performance metrics

Parameter	All identifiedlesions	Lesions ≥5mm	Lesions <5mm
True positive lesions	502	366	136
False negative lesions	105	35	70
False positive lesions	41	12	29
Precision	0.924	0.968	0.824
Sensitivity	0.827	0.913	0.660
F1-Score	0.873	0.939	0.733
Parameter	Median	IQR	Range
Dice Similarity Coefficient	0.771	0.567-0.837	0-0.949

The STU-Net performance in detecting metastatic brain lesions was assessed for all displayed lesions, lesions equal to or larger than 5 mm, and lesions smaller than 5 mm. Moreover, the 3-dimensional segmentation performance of the model is outlined.

### PACS-Based AI-LTT-Facilitated Efficiency Gains

**Figure 2:**
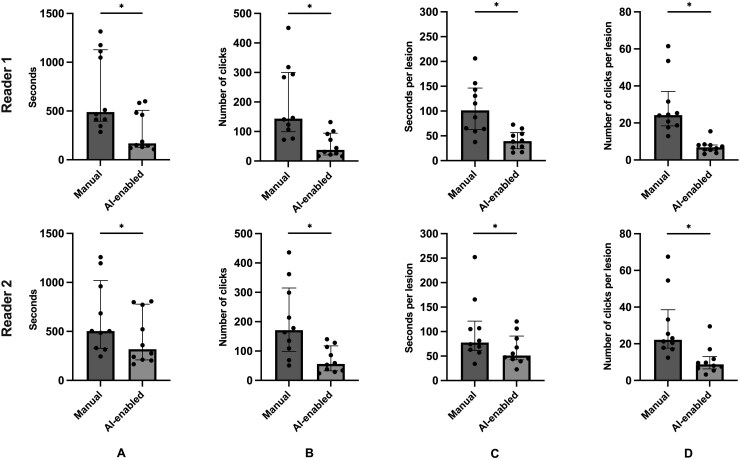
Comparison of manual versus AI-LTT-assisted measuring and tracking of BMs by R1 and R2. (A and B) Per patient examination, the time and necessary number of mouse clicks from opening up to 8 studies until completion of lesion measurements were recorded. (C and D) The seconds and number of mouse clicks were correlated with the maximum number of lesions of each patient. Note that the individual AI-enabled number of clicks showed a close clustering whereas the related numbers for the manual workflow showed a large spread across both readers. This suggests that the AI-enabled measurements may provide a more consistent workflow experience. *P < 0.05.

In completing the entire manual workflow, the median time and number of mouse clicks were 489s [395-1128] and 144 clicks [99-300] for Reader 1. In the AI-assisted workflow, the median time and number of clicks were 168 s [128-507] (*P* = 0.002) and 38 clicks [21-94] (*P* = 0.002) for Reader 1. For Reader 2, completing the manual workflow required a median of 504s [327-1020] and 171 mouse clicks [99-315]; with the AI-assisted workflow, the median time and number of clicks were reduced to 317 [210-778] (*P* = 0.002) and 56 clicks [33-117] (*P* = 0.002), respectively (Figure 2).

In a sub-analysis investigating the efficiency gains separately for the loading and displaying of the studies, the tool reduced the median time for Reader 1 from 42 s [34-52] to 30 s [26-38] (*P* = 0.01) and the number of mouse clicks from 9 [9-12] to 8 [6-9] (*P* = 0.008). In the process of BM measurement, the median time was reduced from 451 s [345-1091] to 138 s [97-475] (*P* = 0.002) and the median number of clicks was reduced from 135 [87-291] to 29 [13-86] (*P* = 0.002) for Reader 1 (see Supplementary Material).

### Inter-Observer Variability

**Figure 3:**
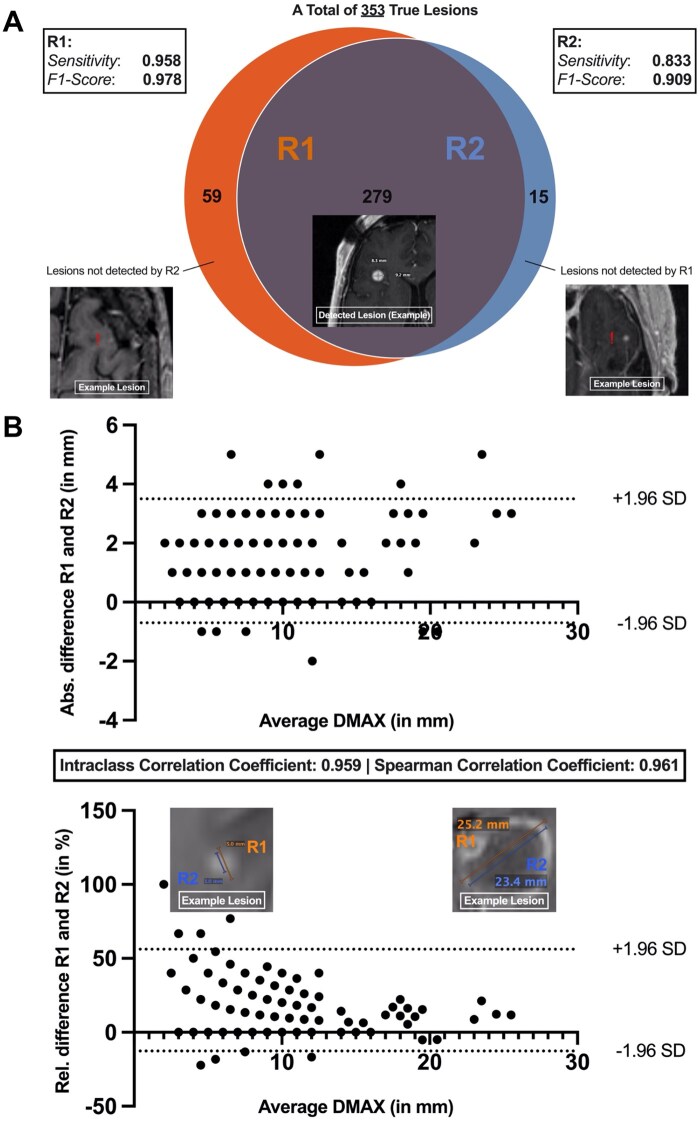
Inter-rater variability in detecting and measuring brain metastases. (A) Based on the 2-dimensional measurements performed by R1 and R2 in the manual workflow, the inter-observer variability in detecting 353 cerebral metastases was analyzed. In addition, examples of lesions that were identified by only one of the 2 readers are displayed. (B) The absolute and relative differences of the 2D measurements performed by R1 and R2 are visualized, including example images of measured lesions. Note that the relative difference of the 2D measurements decreases with increasing lesion size.

**Figure 4:**
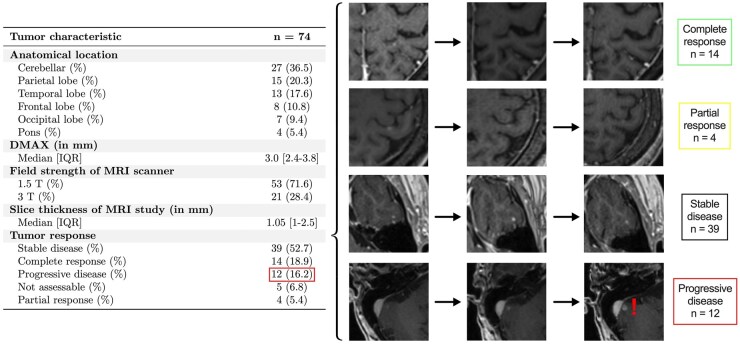
Characterization of discrepant brain metastasis with imaging of example lesions. A total of 74 lesions that were detected by only one of the 2 readers are characterized. For the treatment response, example images showing the behavior of representative lesions over multiple time points are outlined.

We assessed the inter-rater variability between the measurements performed by R1 and R2 in the manual workflow without AI assistance, augmented with the final assessment by a third neuroradiologist who reviewed the discrepant lesions. Among 353 BMs in total, 279 were identified by both R1 and R2; R1 demonstrated a precision of 1 and a sensitivity of 0.958, while R2 showed a precision of 1 and a sensitivity of 0.833 in lesion detection. In 2D measurement of BMs identified by R1 and R2, an intra-class correlation coefficient of 0.959 and a Spearman correlation coefficient of 0.961 were revealed. The relative difference of the 2D measurements decreases as lesion size increases. Subset analysis of 74 discrepant BMs, that were detected by either R1 or R2 but not by both, showed that 36.5% were in the cerebellum, and the median DMAX was 3.0 mm [2.4-3.8]. Importantly, 12 out of 74 BMs exhibited a size increase of more than 20% on follow-up, indicating progressive disease (Figures 3 and 4).

## Discussion

Advances in neuro-oncological care of patients with brain metastases resulted in a growing workload for neuroradiologists for interpretation of BM MRI studies due to longer patient follow-up times, increased complexity of imaging with increased number of followed lesions at different stages of treatment, and the need to lesion-based follow-up to optimize clinical care.^1^^,^^2^^,^^5^^,^^13^ Therefore, there is a critical need to develop automated workflows that allow detection and volumetric measurement of lesions over time of treatment, provide ability by radiologists to modify the lesion outlines used for measurement, and that are standardized across institutions.

Our study presents a prototype of a PACS-integrated AI-driven lesion tracking tool for BM that enables longitudinal monitoring of BM patients using an implemented segmentation network whose accuracy was demonstrated on a 1- and 3-dimensional level. The clinical utility and significant workflow efficiency enhancements of the AI-LTT prototype, which organizes and standardizes BM measurement and reporting, were underscored by involving 2 experienced board-certified neuroradiologists. This tool is a prototype and not a commercial product, therefore development and implementation of vendor neutral tools similar to this tool is currently for research purposes only. Moreover, our results reveal a novel observation of significant inter-rater variability of manual BM measurements of the included expert readers in a controlled setting over a wide range of lesion sizes, with one of the unexpected areas being lesion detection. This presents an opportunity for AI to be used in the standardization of BM measurements to achieve consistent results for precision-based BM treatment approaches across different health systems and infrastructure.

Multiple segmentation algorithms for the enhancing tumor core of pre- and post-treatment BMs are available in the literature. While we used an STU-Net model for this purpose, there are different other network architectures available that also provide accurate BM segmentation which can be used for incorporation into clinical workflow.^20–25^ Based on the observed Dice Similarity Coefficients, the segmentation performance of our STU-Net model (median patient-wise DSC of 0.771) seems to be slightly lower than published algorithms but higher than the results of the BraTS-METS 2023 challenge, particularly in lesion detection^16–18^^,^^26–31^ (Table 2). Showing an interquartile range of 0.567-0.837, the model’s 3D segmentation performance appears variable, which may be due to the wide range of lesion sizes in the used dataset (Table 1). Zhou et al. introduced a deep learning-based 2 stage BM segmentation algorithm in T1 post-gadolinium MRIs that contains a single-shot detector for lesion identification and an FCN for segmentation. This MetNet model showed a mean lesion level DSC of 0.81.^18^ Another BM segmentation network for contrast-enhanced T1-weighted MRIs in SRS settings investigated by Liu et al. applies modern clustering and active contour techniques to achieve a mean lesion-level DSC of 0.89.^27^ However, most of the proposed algorithms require significant pre-processing, such as skull-stripping, which may disrupt the workflow.^14^^,^^16^^,^^18^^,^^26–31^ In contrast, our segmentation pipeline operates on real-world MRIs within PACS, eliminating the need for external preprocessing or data exchange between multiple software systems. We also validated our algorithm on lesions <5 mm as sub-centimeter brain tumors regularly undergo irradiation in clinical practice.^14^^,^^28^^,^^32^ Nevertheless, the AI-LTT-integrated algorithm provides reliable detect-ion and spatial segmentation of BMs, particularly in lesions ≥5 mm that are considered measurable per RANO-BM criteria.^8^

We enhanced the previous version of the lesion tracking tool that Cassinelli Petersen et al. reported by leveraging AI. Assessment of BMs on a lesion-based level was performed, which is a more comprehensive approach than the current recommendation by RANO-BM to use the sum of all measured diameters.^11^^,^^19^ In accordance with the findings of our previous study, the custom hanging protocol and the interactive LTT layout provided significant efficiency gains across 2 readers involved in the present study. However, most of the efficiency improvements were facilitated by the integrated auto-segmentation feature, which may decrease the workload and burn-out rate of physicians in clinical routine^13^ (see Supplementary Material). Based on the significant reduction of time and number of mouse clicks needed for measurement, we infer that this type of a tool could also reduce the cognitive burden for neuroradiologists interpreting BM follow-up studies. When using automated lesion tracking tool in clinical practice, the user verifies the AI-predicted measurements by scrolling through the images instead of measuring all metastatic tumors manually (Figure 2).

AI-driven frameworks for longitudinal BM measurement in post-SRS settings have been reported in the literature.^16^^,^^18^^,^^33–35^ Hsu et al. presented the METRO process, which automatically 3D segments, matches, and tracks BMs and the related radiation doses per lesion over time. This tool also provides 2-dimensional measurements and a final report document containing the automated lesion assessment. The model detected 72% of the new or unirradiated lesions correctly, and a DSC of 0.76 ± 0.03 and an *R*^2^ of 0.80 (AI-generated DMAX validation) were revealed. While this study showed better performance than our algorithm, this study included only 161 lesions and did not demonstrate the clinical utility of the METRO process, conducting no experiments to reveal potential efficiency gains in clinical practice.^36^ Jalalifar et al. and Chitphakdithai et al. proposed similar approaches to track BMs, but these methods require comprehensive preprocessing, such as voxel intensity normalization or skull-stripping, which significantly hinders their translation into clinically usable applications.^34^^,^^37^ The introduced framework of Karami et al. can automatically segment BMs on several follow-up MRI studies post-SRS but does not provide any organization of the measured lesions and was validated using only 5 consecutive patients.^33^ SimU-Net—a simultaneous multi-channel 3D model—uses matching pairs of 3D patches from one pre- and one post-SRS T1 post-gadolinium MRI as input and allows automated detection, segmentation, and matching of BMs and classifies their size changes at 2 different time points. The proposed pipeline using 3 different U-Net models yielded a precision of 0.76 ± 0.27 and sensitivity of 0.94 ± 0.16 in detecting lesions and a mean lesion-wise DSC of 0.87 ± 0.14 in segmenting BMs.^35^ None of the described studies conducted experiments that demonstrate potential efficiency gains in clinical practice facilitated by these tools, and there is no evidence of the clinical utility and translatability of these approaches. Only the models presented by Hsu et al. and Hammer et al. provide an organization of the longitudinal BM lesion assessment in the form of output reports.^33–37^

In our study, 2 neuroradiologists measured metastatic brain tumors on real-world MRI scans on the same PACS interface that is used clinically and deployed the AI-LTT within PACS, thus establishing a paradigm for how BM segmentation algorithms can provide value in clinical practice. Similar to the previous version of our PACS-integrated LTT, the electronic Physician Annotation Device (ePAD) and the Lesion Tracker introduced in 2014 and 2015 also facilitate organized lesion-level-based longitudinal assessment of imaged tumors and standardization of reporting measurements in radiological imaging studies. Potential reader efficiency improvements through the deployment of ePAD were revealed (average time savings of 15%) while the Lesion Tracker seems to enhance the measurement reporting efficiency especially when investigating patients with multiple follow-up images. Yet, these tools still require comprehensive manual user input as there is no integrated automatization available and ePAD is not clinical software.^19^^,^^38^^,^^39^

In contrast to prior studies on automated segmentation and tracking of brain metastases over time, our work uniquely evaluates a PACS-integrated tool operating on real-world clinical imaging data bypassing laborious pre-processing. While earlier approaches typically focused on algorithmic performance alone, our work combines automated segmentation, lesion matching, and longitudinal organization within a single workflow-ready system. Importantly, we included 2 board-certified neuroradiologists to evaluate usability and integration into clinical reading environments—an aspect largely absent in the literature. The utilization of a research PACS, a digital twin of the clinical PACS, further ensures direct translatability into clinical practice.

Inter-observer variability in measuring BMs and other pathologies 2- and 3-dimensionally was demonstrated to be an important feature in the incorporation of measurement metrics into the reporting of brain tumors. In AI-driven segmentation, radiologist segmentations are often treated as the ground truth, yet inter-rater variability and the degree of consensus overlap are typically underexplored. Developing a structured method for defining ground truth could provide a more rigorous foundation, particularly for training AI algorithms. This represents a promising area for further research.^10–12^

Bauknecht et al. revealed mean absolute and relative differences for the maximum diameter measurements of 355 lesions performed by 2 radiologists that ranged between 0.2 and 0.3 mm and between ± 26.8% and ± 33%.^10^ In addition to differences in measuring lesion diameters observed in our study, there were significant differences in the detection of BMs, particularly below 5 mm, which could lead to either under-detection or overcalling of lesions and thus affect patient outcomes (Figures 3 and 4). This inter-rater variability in lesion detection and diameter measurement substantiates the critical need for standardization of BM measurements among different readers. AI-based automatization of measurements has the potential for reducing inter-rater variability in measurements and thus providing more precise measurements in lesion-based follow-up of patients with BM.

### Limitations

Our study was limited by its retrospective character. A planned prospective study could strengthen our current findings regarding the efficiency gains in clinical practice and could help to evaluate the impact of the generated enhanced reports on clinical decision-making. We investigated only existing MRI studies within the 8-viewer LTT interface, whereby the tool also enables prospective assessment of patients as the saved measurements of each patient can be updated with the data of new follow-up scans. We introduced an interval of 10 days between the readers’ manual and AI-assisted measurements to mitigate recall bias. However, the use of the same studies in both workflows introduces potential memory and learning effects that could influence the readers’ measurements within the AI-assisted workflow. In addition, we used 2-dimensional BM measurements, although volumetric assessment has been demonstrated to present a more sensitive measure with less variability.^10^^,^^40^ We chose the diameter-based BM measurements because they represent the current standard of care, while 3D measurements are only optional. Other models that segment brain metastases have been shown to segment the necrotic portion of the tumor or edema surrounding the tumor, which we plan to implement in future generations of the tool.^30^

## Conclusion

We present an AI-driven approach to standardize, organize, and report automated BM measurements using enhanced reports to communicate with treating neuro-oncologists. We also show significant inter-observer variability in BM measurements performed by 2 board-certified neuroradiologists, particularly on lesions smaller than 5 mm. Among the lesions that were not detected by one of the board certified neuroradiologists in manual workflow without AI assistance, 16.2% developed progression. AI-augmented lesion tracking and organization within PACS (AI-LTT) was shown to improve the workflow efficiency for lesion-based assessment of BM MRI workup with multiple prior comparison scans. This clinically usable PACS-integrated tool provides workflow acceleration, facilitated by the embedded custom hanging protocol, auto-segmentation feature, and interactive organizational layout. In accordance with the individual lesion-level treatment provided by SRS, our introduced prototype for AI-assisted lesion tracking within clinical PACS enables lesion-level-based longitudinal disease assessment for all lesion sizes, which offers efficient treatment and surveillance tailored to each BM patient in clinical practice.

## Supplementary Material

vdag036_Supplementary_Data

## Data Availability

The deidentified training dataset used in this study is publicly available at https://doi.org/10.7937/6be1-r748. The images selected for validation are part of an open-access dataset available under https://www.cancerimagingarchive.net/collection/yale-brain-mets-longitudinal/. The algorithm presented in this study can be shared with researchers upon reasonable request. Requests should be directed to the corresponding author.
