# Supplementary figures and images for: Automatic longitudinal assessment of brain metastases improves detection of disease progression
Source: Neurooncol Adv. 2026 Feb 11;8(1):vdag036. doi: 10.1093/noajnl/vdag036 (PMC13082478; doi:10.1093/noajnl/vdag036)

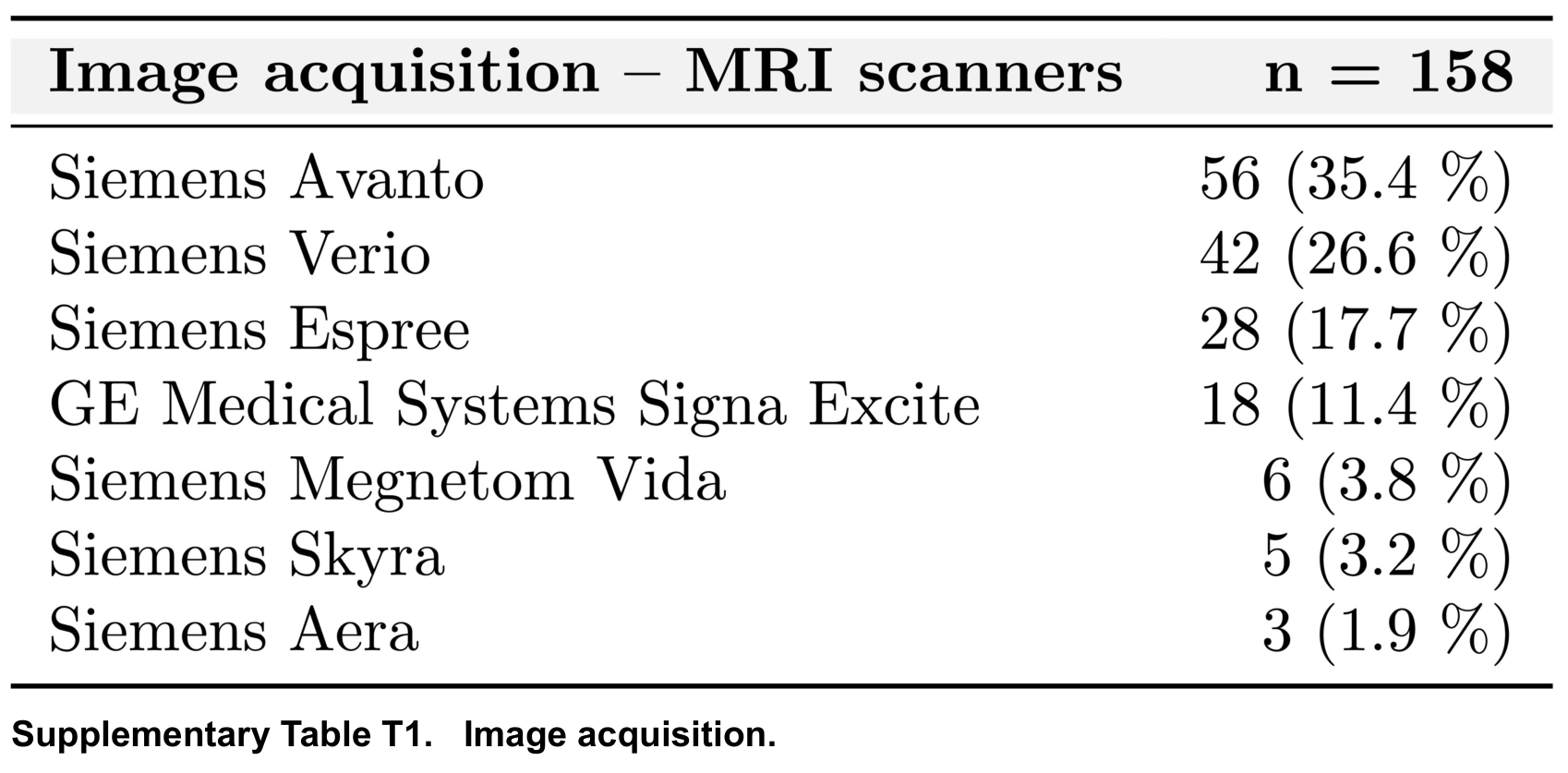

Supplement: vdag036_Supplementary_Data [file vdag036_supplementary_data.zip › AILTT_T1.tiff]

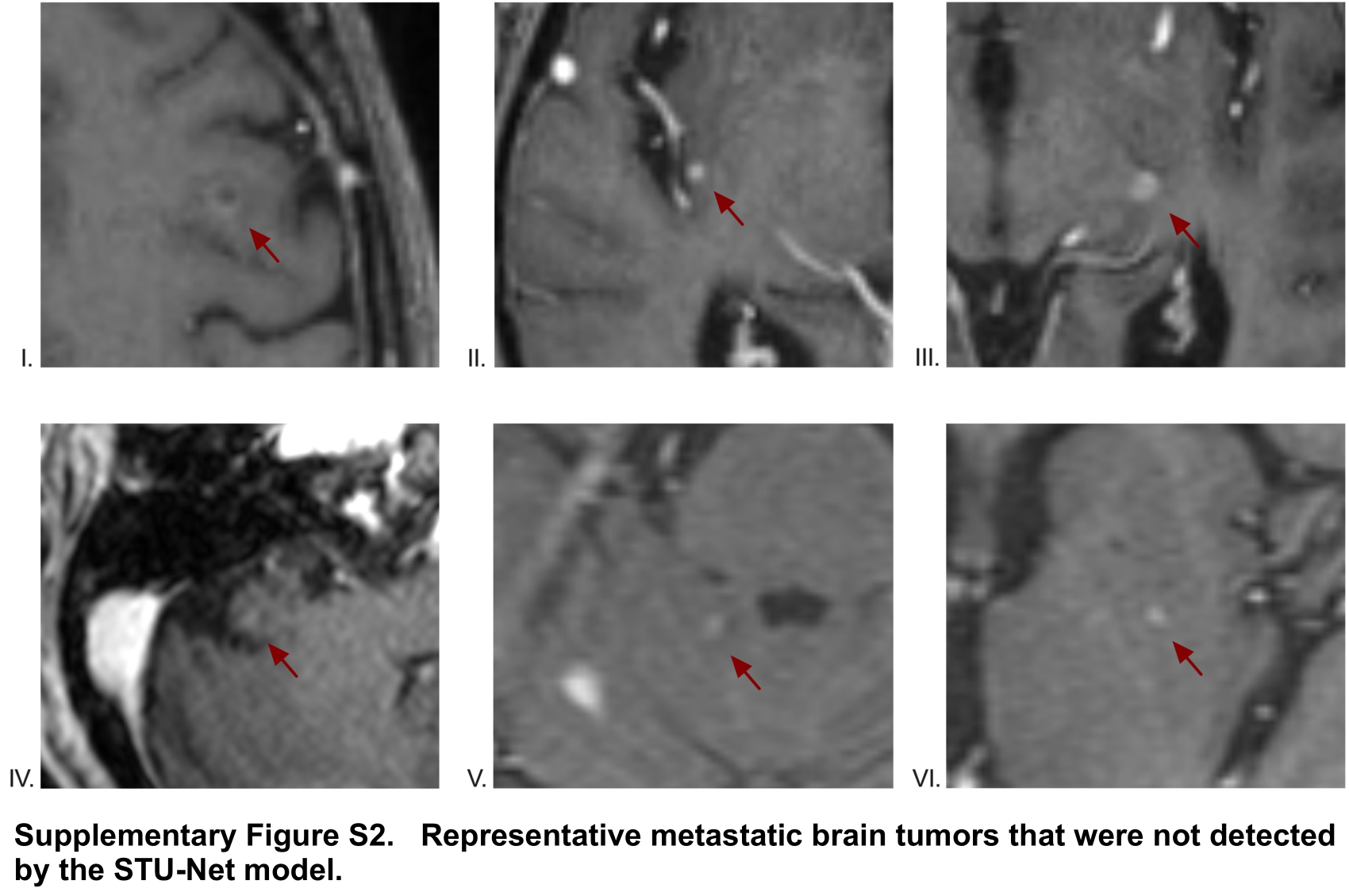

Supplement: vdag036_Supplementary_Data [file vdag036_supplementary_data.zip › AILTT_S2.tiff]

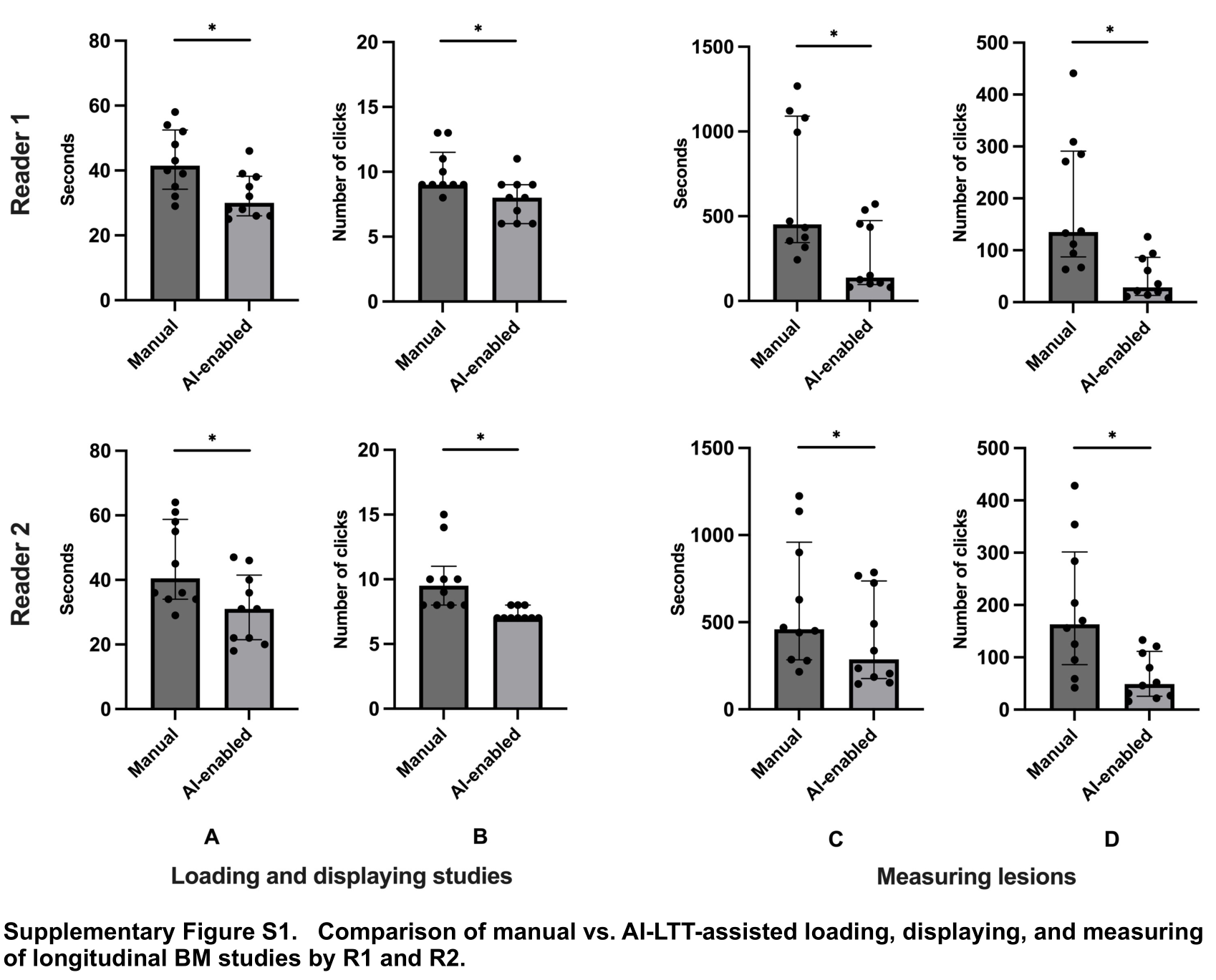

Supplement: vdag036_Supplementary_Data [file vdag036_supplementary_data.zip › AILTT_S1.tiff]
